# Extra-Hippocampal Subcortical Limbic Involvement Predicts Episodic Recall Performance in Multiple Sclerosis

**DOI:** 10.1371/journal.pone.0044942

**Published:** 2012-10-08

**Authors:** Robert A. Dineen, Christopher M. Bradshaw, Cris S. Constantinescu, Dorothee P. Auer

**Affiliations:** 1 Division of Radiological and Imaging Sciences, University of Nottingham, Nottingham, United Kingdom; 2 Department of Psychopharmacology, University of Nottingham, Nottingham, United Kingdom; 3 Division of Clinical Neurology, University of Nottingham, Nottingham, United Kingdom; University of Alberta, Canada

## Abstract

**Background:**

Episodic memory impairment is a common but poorly-understood phenomenon in multiple sclerosis (MS). We aim to establish the relative contributions of reduced integrity of components of the extended hippocampal-diencephalic system to memory performance in MS patients using quantitative neuroimaging.

**Methodology/Principal Findings:**

34 patients with relapsing-remitting MS and 24 healthy age-matched controls underwent 3 T MRI including diffusion tensor imaging and 3-D T1-weighted volume acquisition. Manual fornix regions-of-interest were used to derive fornix fractional anisotropy (FA). Normalized hippocampal, mammillary body and thalamic volumes were derived by manual segmentation. MS subjects underwent visual recall, verbal recall, verbal recognition and verbal fluency assessment. Significant differences between MS patients and controls were found for fornix FA (0.38 vs. 0.46, means adjusted for age and fornix volume, *P*<.0005) and mammillary body volumes (age-adjusted means 0.114 ml vs. 0.126 ml, *P*<.023). Multivariate regression analysis identified fornix FA and mammillary bodies as predictor of visual recall (R^2^ = .31, *P* = .003, *P* = .006), and thalamic volume as predictive of verbal recall (R^2^ = .37, *P*<.0005). No limbic measures predicted verbal recognition or verbal fluency.

**Conclusions/Significance:**

These findings indicate that structural and ultrastructural alterations in subcortical limbic components beyond the hippocampus predict performance of episodic recall in MS patients with mild memory dysfunction.

## Introduction

Cognitive impairment occurs in 30%–70% of multiple sclerosis (MS) patients and commonly manifests as disturbances in memory, complex attention, efficiency of information processing, executive functioning, and processing speed [1]. Cognitive impairment in MS impacts on quality of life, vocational status and social functioning [2], [3], [4]. Impairment of episodic memory is one of the most common cognitive deficits in MS, with particular involvement of recent memory and to a lesser extent immediate and remote memory [5]. Both visual and verbal memory functions are affected being impaired in 56% and 34% of MS patients respectively [6]. However, conventional radiological measures of the extent of disease involvement, such as T2 lesion load, remain poor predictors of cognitive impairment in MS [7].

Evidence from animal and human studies has shown that episodic memory is dependent on the extended hippocampal - diencephalic system [8]. This system consists of a series of direct and indirect parallel temporo-diencephalic pathways with damage to individual components leading to impairments in learning and recall, clinically manifesting as anterograde amnesia syndromes [9], [10]. However, familiarity-based recognition is not thought to be dependent on the integrity of this system [9].

A number of studies have used imaging to study the impact of damage of isolated components of the extended hippocampal - diencephalic system on cognitive performance in MS, but there is a lack of studies employing a comparative quantitative approach to identify the relative importance of damage to components of the integrated system. Selective atrophy of the hippocampus has been described in relapsing-remitting multiple sclerosis (RRMS) [11], and this has been found to correlate with performance in tests of verbal learning/recall in MS patients [11], [12]. Atrophy of the thalamus occurs in MS [13], [14], and shows a significant relationship to cognitive performance when measured directly or indirectly [15], [16], [17], [18]. Mammillary body atrophy has also been identified in MS [19], but the cognitive relevance of this finding has not, to our knowledge, been tested.

The fornix is the major efferent pathway from the hippocampus projecting to the mammillary bodies via the post-commissural fornix, as well as numerous other sites including the nucleus accumbens, septal nuclei and prefrontal cortex (via the pre-commissural fornix), the hypothalamus and anterior thalamic nuclei. Three recent studies using tract-based spatial statistics [20] have identified reduced fractional anisotropy (FA, a measure of white matter integrity derived from diffusion tensor imaging, DTI) in the fornices of patients with multiple sclerosis compared to healthy controls [21], [22], [23]. Roosendaal et al report increases in both axial and radial diffusivity within the fornix of MS patients compared to the controls, with the elevation of radial diffusivity being more significant than axial diffusivity [22]. Kern et al have conducted a combined fMRI and DTI study in 18 MS patients and 16 controls and again demonstrated the relationship between structural damage to the fornix an poorer performance in a verbal memory task [24]. Furthermore, they found differences in hippocampal activation during a verbal memory task that were related to fornix FA, interpreted as being a limiting effect of damage to the fornix on disease-related compensatory activity.

The purpose of this study is thus to test the relative contributions of loss of structural integrity in components of the extended hippocampal–diencephalic system to cognitive performance in MS. In particular we aim to identify the relative contribution of reduced hippocampal, thalamic and mammillary body volumes and reduced fornix tract integrity to performance in tests of visual memory/recall, verbal learning and recall, verbal recognition and a non-memory dependent test of verbal fluency. We go beyond our previous whole brain DTI analysis of this cohort [21] and the work of others who have studied integrity of components of this system in MS, by creating regression models using the limbic integrity measures (and including potential confounders) to identify the limbic structures for which reduced integrity most predicts episodic memory performance.

## Methods

### Participants

Thirty-four patients with definite relapsing remitting MS (RRMS) (median age 42.6 years, range 31.1 to 56.1, M∶F = 1∶2.1) were prospectively recruited from a local database of RRMS patients after giving written informed consent. Participants with MS had median disease duration 10 years (range 3 to 27 years) and median Expanded Disability Status Scale (EDSS) of 2.5 (range 1.5 to 6.5) at the time of the study. Participants with MS were not taking immunomodulatory therapy and had not had a relapse or steroid therapy for at least two months prior to inclusion. Twenty-four healthy controls (median age 38.7, age range 28.3 to 55.3, M∶F = 1∶1.7) were included. Participants were excluded if they had any other significant neurological, medical, psychiatric or cognitive disorder. Analyses based on this participant cohort have been reported in a previous publication [21].

### Neuropsychological testing

All MS patients underwent standardized neuropsychological testing by a single researcher on the day of the MRI scan. The tests performed were the Benton Visual Retention Test (BVRT), a test of visuospatial memory [25], California Verbal Learning Test version II (CVLT-II), a test of verbal memory [26] and Controlled Oral Word Association Test (COWAT), a test of verbal fluency [27]. CVLT-II subscores used were the short delay free recall (CVLT-II recall) and the total recognition discriminability (CVLT-II recognition). The National Adult Reading Test (NART) was performed in all MS subjects and used to estimate pre-morbid IQ (WAIS FS-IQ) [28]. Unadjusted scores or Z-transforms (based on published datasets [26], [29], [30]) were used as described previously [21].

### MRI acquisition and image processing

All subjects underwent MRI scanning at 3 Tesla (Achieva, Philips, Eindhoven, NL), including axial DTI (Single-shot diffusion weighted EPI, b = 1000 s/mm^2^, 15 directions, TE = 56 ms, TR = 9700 ms, 2 mm×2 mm×2.5 mm voxel size interpolated to 1 mm×1 mm×2.5 mm voxels, 45 interleaved slices with no gap, four averages), sagittal MPRAGE (TR = 7.5 ms, TE = 2.2 ms, Flip = 8, Matrix 256×256, voxel size = 0.8 mm×0.8 mm×0.8 mm, TFE factor = 236, FOV = 205), and axial FLAIR (TR = 11 000 ms TE = 125 ms TI = 2800 ms, matrix 256_256, slice thickness 2.5 mm/0 mm gap, FOV = 256). DTI data underwent eddy current correction and removal of non-brain voxels [31], and was further post-processed using the fMRIB diffusion toolbox (FSL version 3.3 [32]), to give individual subject maps of FA, axial diffusivity (AD) and radial diffusivity (RD).

#### Volumetric measurements (Figure 1)

**Figure 1:**
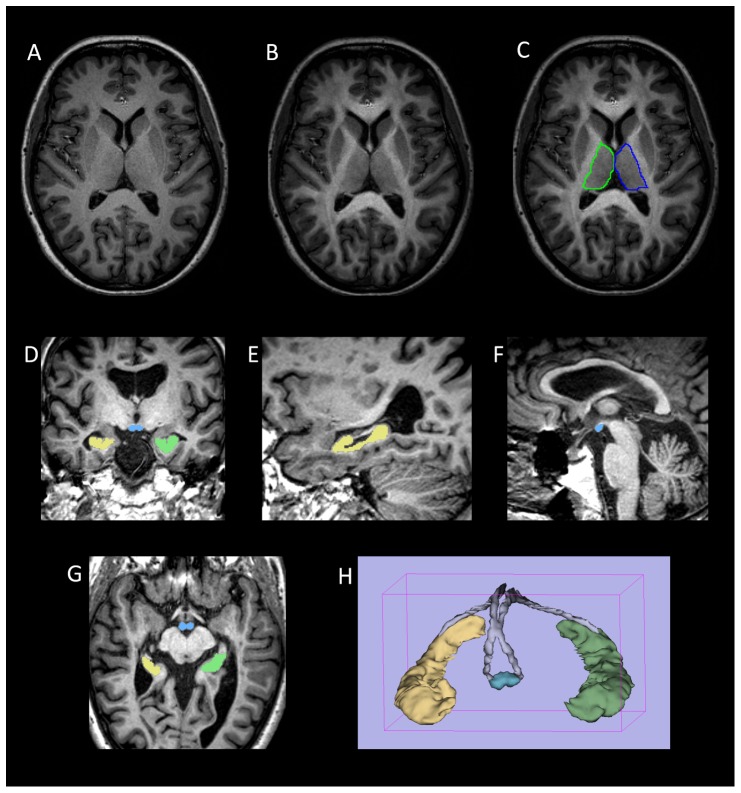
Volumetric segmentation of brain images. (a–c) Thalamic segmentation. Axial MPRAGE image from a control subject (a) before and (b) after the addition of FA weighting from the co-registered FA image. Note the increase in clarity of the lateral border of the thalamus that facilitates (c) manual segmentation of the thalamus. (d–g) axial, coronal and sagittal images from a participant with MS demonstrating ROIs drawn for the mammillary bodies (blue) and right (yellow) and left (green) hippocampi. (h) Surface rendered representation of the segmented hippocampi and mammillary bodies from a participant with MS. The manually defined fornix ROI used to derive fornix FA is also shown (grey).

Semi-automated brain extraction of the MPRAGE images was performed using the Brain Finder tool within JIM 3.0 software (Xinapse Medical Systems, UK) to provide total intracranial volume (TICV) and brain parenchymal volume, from which brain parenchymal fraction (BPF) was derived (BPV/TICV).

Volumes of the hippocampi, mammillary bodies and thalami were obtained manually from the MPRAGE images using 3D-Slicer software (MIT, Cambridge, MA, USA). The measurements were made by a single trained academic neuroradiologist (RAD) blinded to disease, cognitive and demographic status. Anatomical boundaries of the hippocampus were defined according to Pantel and co-workers [33]. The mammillary bodies were outlined using axial, sagittal and coronal planes according to previously described boundaries [34].

The thalami were outlined in the axial plane, commencing on the slice at the level of the anterior commissure (below which reliable separation of the thalamus from subthalamic structures is difficult) and proceeding dorsally to the most dorsal slice on which the thalamus could clearly be visualized. To aid visualization of the lateral edge of the thalamus (which merges with the internal capsule on MPRAGE images), MPRAGE images were weighted by FA as follows: The subjects FA map was first coregistered to the MPRAGE using FSL FLIRT, following which the accuracy of the coregistration was checked by careful visual inspection. The MPRAGE images were then multiplied by (1+FA) to give the FA-weighted MPRAGE image. The high FA in the internal capsule provides a clear position of the lateral thalamic boundary, which aided the manual thalamic outlining (Figure 1 a–c). Hippocampal, mammillary body and thalamic volumes were normalized to head size according to the analysis of covariance approach proposed by Jack Jr et al [35].

Hippocampal, thalamic and mammillary body volumes were re-measured in a subset of 10 MS patients (thus 20 of each structure) after an interval of at least one week to allow assessment of intra-observer reliability (Type A intraclass correlation coefficients using an absolute agreement definition). Inter-observer reliability was performed in a similar way, with the repeat measurements made by a second trained researcher.

#### Derivation of the fornix FA

The Fornix ROI was drawn manually onto the MPRAGE images as follows. Using the sagittal plane, a marker was placed at the point where the fimbria-fornix detached from the hippocampal tail to become the fornix proper. This marker was then identified in the axial plane as the commencement point for the fornix ROI. The ROI was then drawn to include the crus of the fornix using successively more dorsal slices, before switching to the coronal plane to continue the ROI rostrally through the bodies of the fornix. The axial plane was then used to define the ROI down the fornix columns, terminating at a point immediately dorsal to the mammillary bodies. Care was taken to restrict the ROI to voxels within the fornix, with exclusion of voxels lying on the margin of the fornix which would be prone to CSF contamination due to partial volume effects. Both left and right sides of the fornix were defined in this way, resulting in a single fornix ROI that included left, right and midline fornix components. Finally, the placement of the ROI was checked the axial, sagittal and coronal planes, and edited as appropriate. The ROI was then used to extract mean fornix FA, AD and RD from the coregistered DTI image, and to calculate the fornix ROI volume.

**Figure 2:**
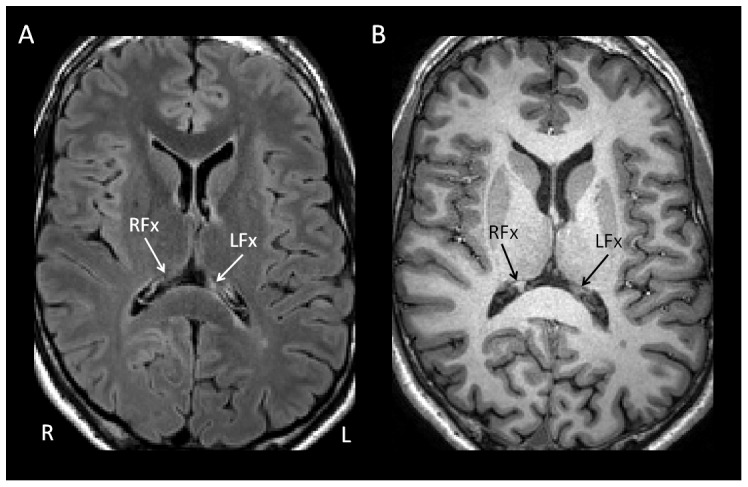
Identification of focal MS lesions of the fornix. (a) Axial FLAIR image from one of the participants with MS showing focal T2-hyperintesity in crus of the left fornix (LFx) with accompanying T1-hypointensity on the corresponding MPRAGE image (b). Crus of the right fornix labelled for comparison (RFx).

In addition, the FLAIR images were carefully inspected for the presence of focal T2 hyperintense lesions. When focal T2 hyperintensity was detected in the fornix, the relevant MPRAGE images were inspected for corresponding T1, which was found to help confirm the presence of a genuine focal lesion (Figure 2).

### Ethics

The study was approved by the UK National Health Service Research Ethics Committee (Nottingham 1 Committee), and all participants in this study gave written informed consent.

### Statistical analysis

Statistical analysis was performed using IBM SPSS Statistics 19.0 (IBM Corporation. Somers, NY). A significance threshold of α = 0.05 was used, unless stated otherwise. Normality of data distribution was assessed by the Shapiro-Wilk test prior to use of parametric tests. Comparison of age between the MS patients and controls was made using Independent-Samples T-Test. Between group comparisons of normalized hippocampal volumes, normalized thalamic volumes and normalized mammillary body volumes were performed using analysis of covariance (ANCOVA) treating group as a fixed factor and age as a covariate. Group comparisons of forniceal ROI FA, AD and RD values were performed using ANCOVA with age and fornix volume included as covariates, the latter included to account for account for partial volume edge effects on the FA measures [36]. Group comparison was also planned to test whether fornix FA differed in those with and without focal T2 hyperintense lesion in the fornix.

To test for independent contributions of structural and ultrastructural alterations on neuropsychological performance we carried out standard multivariate stepwise regression treating the neuropsychological scores as the dependent variable. The main imaging variables of interest (fornix FA and normalized hippocampal, thalamic and mammillary body volumes), along with age, brain parenchymal volume and estimated premorbid IQ (considered as potential confounding variables), and normalized fornix volume (included to control for partial volume effects of CSF signal on fornix DTI measures) were included. The model used entry and exit criteria of *P*<0.05 and *P*<0.10 respectively. The regressions were repeated with fornix AD and RD substituted in place of fornix FA.

## Results

### Comparison of MS patients and controls

**Figure 3:**
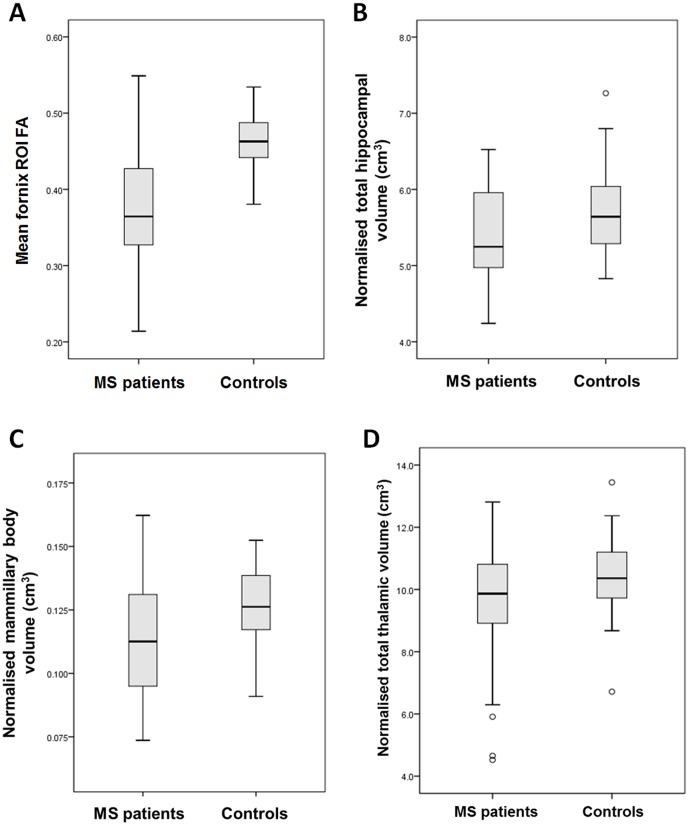
Boxplots showing mean (a) mean fornix FA, (b) normalized total hippocampal volumes, (c) normalized total mammillary body volumes and (d) normalized total thalamic volumes for the MS and control groups.

The difference in age between MS patients and controls was not statistically significant (42.6 years vs 38.7, *P* = .07). Mean fornix FA was strongly reduced in MS patients vs. controls (means adjusted for age and normalised fornix volume: −0.37 vs 0.46, *F* (1,55) = 34.6, *P*<.0005, Figure 3a). Age was a significant covariate in the ANCOVA model (*F* (1,55) = 5.7, *P* = .021), but normalised fornix volume was not (*P* = .062). A group difference was also identified for mean fornix RD (means adjusted for age and normalised fornix volume: 0.00129 vs 00100, *F* (1,55) = 27.8, *P*<.0005), but not for mean fornix AD.

Intra- and inter-observer reliability measures of volumes derived by manual segmentation were good (table 1). The group difference in normalized hippocampal volume fell just outside the predefined significance threshold (age-adjusted means: 5.38 ml vs 5.71 ml, *F* (1,56) = 3.8, *P* = .057, Figure 3b). Age was not a significant covariate in the ANCOVA model (*P* = .59). A significant group difference was found for normalized mammillary body volumes (age-adjusted means: 0.114 ml vs 0.126 ml, *F* (1,56) = 5.5, *P* = .023, Figure 3c). Age was not a significant covariate in the ANCOVA model (*P* = .12). Normalized thalamic volumes were not different between MS patients and controls (age adjusted means: 9.53 ml vs 10.36 ml, *P* = .10) (Figure 3d).

**Table 1:** Intra- and inter-observer reliability assessment for the volumetric measurement, made in a subset of 10 MS patients (thus 20 of each structure).

	Intra-observer reliability			Inter-observer reliability		
Structure	Intraclass correlation coefficient*	95% confidence intervals	Significance	Intraclass correlation coefficient*	95% confidence intervals	Significance
Hippocampal volume	0.93	0.43–0.98	P<0.0005	0.86	0.67–0.94	P<0.0005
Thalamic volume	0.96	0.71–0.98	P<0.0005	0.83	0.17–0.94	P<0.0005
Mammillary body volume	0.92	0.78–0.97	P<0.0005	0.83	0.54–0.94	P<0.0005

* Type A intraclass correlation coefficients using an absolute agreement definition.

### Neuropsychological test performance (table 2)

**Table 2:** Neuropsychological test Z-scores (mean and range) for the MS cohort.

				One sample T-test*	
Test	n	Mean Z score	Z-score range	t	p
BVRT	34	−0.35	−3.1 to 2.1	−1.48	n/s
CVLT-II recall	34	−0.16	−2.5 to 1.5	−0.81	n/s
CVLT-II recognition	34	0.02	−3.0 to 1.5	0.08	n/s
COWAT	34	−0.56	−3.8 to 1.7	−2.78	0.009

* against normative mean value of 0.

The MS subjects showed no impairment of estimated premorbid intelligence (WAIS FS_IQ score mean = 102.5, range = 81 to 118, s.d. = 10, as derived from NART). Mean neuropsychological scores fell within one standard deviation of the normative mean in keeping with a minimal overall neuropsychological burden, although the cohort included individuals with a range of performance from above to more than two standard deviations below the normative mean for each test.

### Relationships between fornix FA and normalized hippocampal, mammillary body and thalamic volumes

Considering all subjects, fornix FA correlated with thalamic volumes (*R* = .45, *P*<0.0005), but not with hippocampal volumes. When subjects with MS were considered separately, correlation was found between fornix FA and normalized thalamic volumes (*R* = .55, *P*<0.0005), but no other correlation was found.

**Figure 4:**
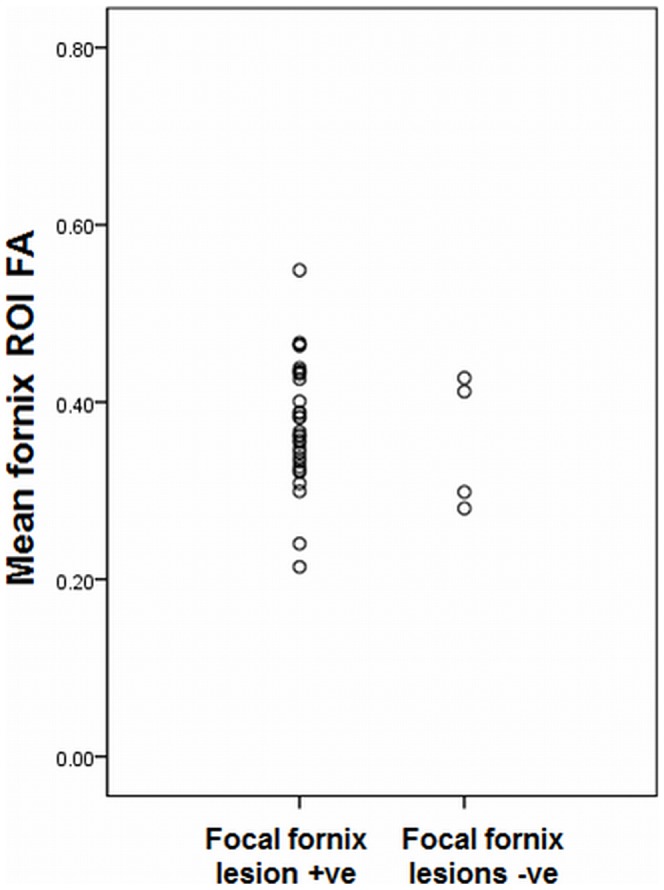
Scatterplot showing mean fornix FA for the MS participants with and without visible T2 hyperintense lesions in the fornix.

It was not possible to formally test whether fornix FA differed in those with and without focal T2 hyperintense lesion in the fornix, as only four MS subjects had a confirmed T2 hyperintense lesion, but the scatterplot of FA for these two groups shows no clear difference in fornix FA between these groups (Figure 4).

### Regression analysis of local and global imaging-derived measure for neuropsychological performance (Table 3)

**Table 3:** Results of the regression analysis for performance in the four neuropsychological tests.

	Final regression model		Unstandardized coefficients			
Neuropsychological test	R^2^	Variables retained	B (95% CI)	Standard error	Standardized coefficient (β)	Significance (*P*)
BVRT	**0.31**	**Fornix FA**	12.24 (3.44–21.04)	4.32	**0.42**	**.008**
		**nMBV**	0.30 (0.00–0.06)	0.14	**0.32**	**0.038**
		(constant)	−1.09 (−5.50–3.32)	2.16	-	.618
CVLT-II recall	**0.37**	**nTV**	0.95 (0.50–1.39)	0.22	**0.61**	**<.0005**
		(constant)	1.70 (−2.62–6.01)	2.12	-	.428
CVLT-II recognition	**0.18**	**BPF**	7.88 (1.42–14.34)	3.16	**0.42**	**.019**
		(constant)	−3.63 (−9.16–1.91)	2.71	-	.191
COWAT	**0.14**	**Estimated premorbid IQ**	0.41 (0.04–0.79)	0.18	**0.37**	**.032**
		(constant)	−7.91 (−46.31–30.49)	18.85	-	.678

Abbreviations: nMBV = normalized mammillary body volume; nTV = normalized thalamic volume; BPF = brain parenchymal fraction; CI = confidence interval.

**Figure 5:**
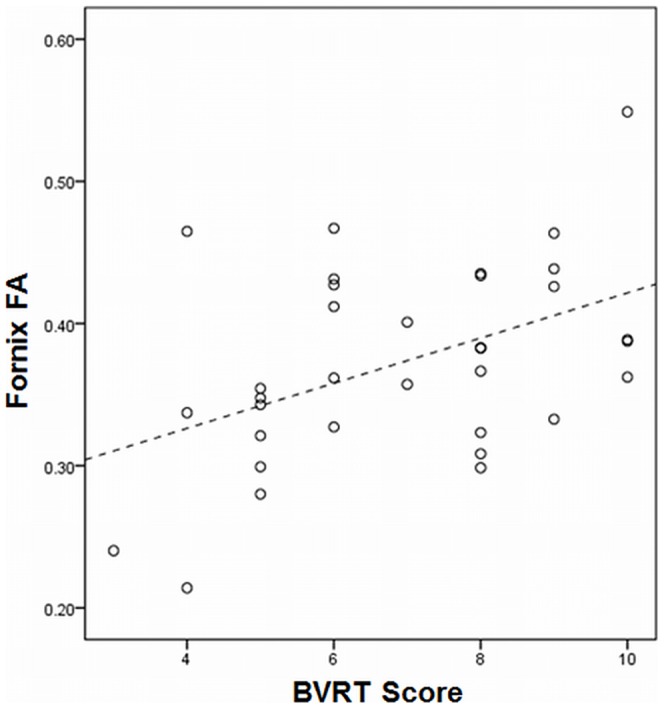
Scatterplot showing the relationship between mean fornix FA and BVRT scores in the MS group.

For BVRT, fornix FA and normalised mammillary body volumes were retained in the model (R^2^ = .31, *P* = .003, Figure 5). For CVLT-II recall, normalized thalamic volume was retained in the final regression (R^2^ = .37, *P*<.0005). For CVLT-II recognition and COWAT scores, the final regression models consisted of only a single non-limbic variable (BPF and estimated premorbid IQ respectively) and accounted for only a small proportion of the variance. Substitution of mean fornix AD or RD in place of fornix FA in the regression did not improve the strength or significance of the regression models for any of the neuropsychological tests.

## Discussion

This study identifies reduced structural integrity in the limbic system beyond the hippocampus as being an important substrate underlying episodic memory performance in MS. Fornix FA and thalamic volume contributed significantly to regression models of episodic memory performance corrected for age and estimated premorbid IQ, whereas global neuroimaging measure of brain atrophy was not predictive. This relationship was not found for verbal recognition or a verbal fluency task. Our data therefore support an anatomical dissociation between visual/verbal recall and verbal recognition, with the latter showing no statistical relationship with fornix and diencephalic integrity. Animal models have shown impaired recall but preserved recognition memory of fornix disruption in animals with fornix lesions [37], [38], [39]. Data from human studies is more limited but a similar pattern of recall impairment with spared recognition has been observed in post-surgical patients with damage to the fornix and mammillary body atrophy [40], [41].

Recent evidence demonstrating that fornix FA is reduced in both Alzheimer Disease (AD) and MCI, and that fornix FA can discriminate between MCI patients and controls [42], is pertinent to the current results; it is possible that the cognitively-relevant fornix FA reduction in the MS patients reflects a shared anatomical substrate for cognitive dysfunction with MCI patients. Furthermore, attention has recently focused on the role of disruption of large scale brain networks in neurodegenerative disorders [43]. In AD, episodic memory and visuospatial functions are affected early in the disease course, and this is known to be associated with disruption of the default mode network (DMN), which includes medial prefrontal cortex, posterior hippocampus, and posterior cingulate cortex/retrosplenial cortex [44], [45]. The DMN has been shown to be altered in MCI patients [46] and those at risk of developing AD [47]. It is possible that the cognitively-relevant fornix FA reduction shown here contributes to altered hippocampal connectivity to other nodes of the DMN. The recent finding by Roosendaal et al that reduced functional connectivity of the hippocampus is detectable in MS patients prior to the onset of measurable hippocampal atrophy [48] supports this notion. Reduced ultrastructural integrity of the fornix provides a potential substrate for the altered hippocampal connectivity but longitudinal studies will be of importance to establish the complex temporal relationships between fornix degeneration, hippocampal atrophy, hippocampal functional connectivity and deterioration in episodic memory performance in MS.

Unlike previous studies, we found no relationship between hippocampal volume and memory performance. As the volume reduction of the hippocampus in the MS patients was modest and did not reach significance, our findings do not necessarily contradict previous studies showing a relationship between hippocampal atrophy and memory function in MS; instead, it is possible that alterations of the fornix and diencephalic structures predominates for mild degrees of cognitive dysfunction in MS. For example, in the study by Sicotte et al which found a relationship between hippocampal volume and verbal memory in MS patients, both the RRMS (n = 23) and SPMS (n = 11) groups showed significant impairment of verbal memory compared to the control group (n = 18). In line with previous studies, we found a relationship between cognitive performance and thalamic volumes [14], [18], although contrary to previously published studies the reduction in normalised thalamic volumes in the current MS cohort did not reach statistical significance.

It is notable that the current analysis did not find a relationship between fornix FA and CVLT-II recall performance while our previous TBSS analysis of this dataset identified clusters in the left fornix where reduced FA correlated with CVLT-II recall performance. This difference presumably relates to the use of a whole fornix ROI including both left and right sides of the fornix in the current study compared to the voxelwise approach employed by TBSS,

The final regression models for visual and verbal recall only accounted for 31% and 21% of variance in performance respectively, thus a large proportion of the variance remains unaccounted for which there are a number of possible explanations. Firstly the measures of integrity chosen for this analysis (fornix FA and limbic subcortical gray matter volumes) may not have sufficient sensitivity to measure subtle alterations in tissue integrity of these structures. Secondly, pre-morbid memory performance is likely to account in part for the variability, and we have attempted to control for pre-morbid cognitive ability using an estimate of premorbid IQ. Thirdly, as shown in our previous analysis of this cohort brain white matter tract integrity outside the limbic system related to both verbal and visual memory performance, and hence extra-limbic brain integrity may also contribute to the unexplained variance in episodic memory performance.

### Limitations

The 34 MS participants had range of cognitive performance levels, from those with normal performance through to those with performance at and beyond 2.5 standard deviations below the normative population mean, but only a small number had evidence of significant cognitive dysfunction (for example, only four had BVRT Z-score ≤−2). It is therefore possible that the observed relationship between episodic memory performance and the imaging metrics of limbic integrity may be contributed to by inherent premorbid variations in these measures between individuals, as opposed to being the result of disease-specific changes in these processes. Indeed, as Rudebeck and colleagues have shown fornix microstructure relates to episodic recall in healthy volunteers [49].

A further limitation that we acknowledge is that there is potential for fornix FA to be altered by partial volume effects from adjacent CSF. We defined the fornix ROI on MPRAGE images and then extracted the DTI metrics from linearly coregistered images. We acknowledge that potential registration errors may arise that could affect fornix diffusion measurements, but have attempted to minimise this by careful inspection of the coregistered images for errors, particularly in the region of the fornix. While many studies reporting DTI metrics do not correct for partial volume effects, we have attempted to control for this by including normalised fornix volume in the statistical analyses that include FA measures. A similar but less anatomically-specific approach has previously been used by Rashid et al, who used whole brain parenchymal volumes to perform a CSF correction on DTI data from MS patients [36]. Recently this approach has been criticised [50] and a more robust partial volume correction using free water elimination has been advocated [51]. However, this approach has not yet been widely implemented in clinical studies.

In summary, the study establishes the contribution of reduced integrity of the fornix and diencephalic limbic structures to episodic memory performance in MS, a finding which is in line with the current literature from animal studies and human studies. This is, to our knowledge, the first study to demonstrate that reduced integrity in these structures plays a predominant role over hippocampal volume loss in episodic memory performance in MS. Our results provide further evidence for the notion that cognitive performance in MS is influenced by a combination of white matter disruption resulting in disconnection phenomena, and grey matter atrophy.
